# The Sulfatase Pathway for Estrogen Formation: Targets for the Treatment and Diagnosis of Hormone-Associated Tumors

**DOI:** 10.1155/2013/957605

**Published:** 2013-02-13

**Authors:** Lena Secky, Martin Svoboda, Lukas Klameth, Erika Bajna, Gerhard Hamilton, Robert Zeillinger, Walter Jäger, Theresia Thalhammer

**Affiliations:** ^1^Department of Pathophysiology and Allergy Research, Center for Pathophysiology, Infectiology and Immunology, Medical University of Vienna, Waehringer Guertel 18-20, 1090 Vienna, Austria; ^2^Ludwig Boltzmann Cluster Translational Oncology, Waehringer Guertel 18-20, 1090 Vienna, Austria; ^3^Department of Clinical Pharmacy and Diagnostics, University of Vienna, 1090 Vienna, Austria

## Abstract

The extragonadal synthesis of biological active steroid hormones from their inactive precursors in target tissues is named “intracrinology.” Of particular importance for the progression of estrogen-dependent cancers is the *in situ* formation of the biological most active estrogen, 17beta-estradiol (E2). In cancer cells, conversion of inactive steroid hormone precursors to E2 is accomplished from inactive, sulfated estrogens in the “sulfatase pathway” and from androgens in the “aromatase pathway.” Here, we provide an overview about expression and function of enzymes of the “sulfatase pathway,” particularly steroid sulfatase (STS) that activates estrogens and estrogen sulfotransferase (SULT1E1) that converts active estrone (E1) and other estrogens to their inactive sulfates. High expression of STS and low expression of SULT1E1 will increase levels of active estrogens in malignant tumor cells leading to the stimulation of cell proliferation and cancer progression. Therefore, blocking the “sulfatase pathway” by STS inhibitors may offer an attractive strategy to reduce levels of active estrogens. STS inhibitors either applied in combination with aromatase inhibitors or as novel, dual aromatase-steroid sulfatase inhibiting drugs are currently under investigation. Furthermore, STS inhibitors are also suitable as enzyme–based cancer imaging agents applied in the biomedical imaging technique positron emission tomography (PET) for cancer diagnosis.

## 1. Introduction

Estrogens play an important role in regulating cell proliferation and apoptosis in cancer cells of hormone-sensitive tumors in the breast, ovary, endometrium, and other various hormone-sensitive tissues, for example, colon. They are also important for the pathogenesis of nonmalignant disease, including the metabolic syndrome and Type 2 diabetes, diseases often associated with a higher risk for certain malignancies.

The biological most active estrogen, 17beta-estradiol (E2), is important for the homeostasis of cellular metabolism and growth. In premenopausal women, most of the E2 is produced by the gonads and functions as a circulating hormone. This is described by the term “endocrinology.” After the menopause, the levels of circulating estrogens are low, and most of E2 is produced from adrenal steroid precursors at extragonadal sites in various organs including breast, brain, liver, bone, and fat. Extragonadal production of estrogens from adrenergic precursors in target tissues is also important in men having low levels of circulating estrogens. In target tissues, estrogen acts locally either in an intracrine or paracrine way. Production of E2 in the tissue where it regulates cellular processes is described by the term “intracrinology” [1]. 

Two pathways are important for the local E2 production in target tissues, namely, the “sulfatase pathway,” in which biological inactive steroid sulfates are the source for E2, and the “aromatase pathway,” in which E2 is derived from androgenic precursors [2].

Estrogens exert many biological effects through binding and activation of nuclear estrogen receptors (ER), ERalpha and ERbeta, as well as through membrane-associated receptors. Activation of genomic and/or nongenomic signaling pathways contributes to the regulation of cell proliferation and differentiation [3]. Estrogens control the production and activity of components in the cell cycle progression, including cyclines, cyclin-dependent kinases, and their inhibitors [4]. Additionally, direct cancerogenic effects of estrogens can occurs via formation of electrophilic, redox-active estrogen ortho-quinones from catechol estrogens. The concurrent formation of reactive oxygen species and superoxide anions can damage DNA and cellular proteins [5].

In serum and tissues like the female breast, estrogens are mainly present in their inactive sulfated form [5, 6]. The important precursor for E2 in the “sulfate pathway” is inactive estrone-3-sulfate (E1S). This is the most abundant estrogen in women at all ages as well as in men. Levels of E1S in blood are 5–10-fold higher than that of unconjugated estrogens, estrone (E1), estradiol (E2), and estriol (E3). As it has also a longer half-life than E2, it is considered as storage form for estrogens in some organs, for example, breast, from where active E1 is liberated by removal of the sulfate through STS [7, 8]. 

**Figure 1:**
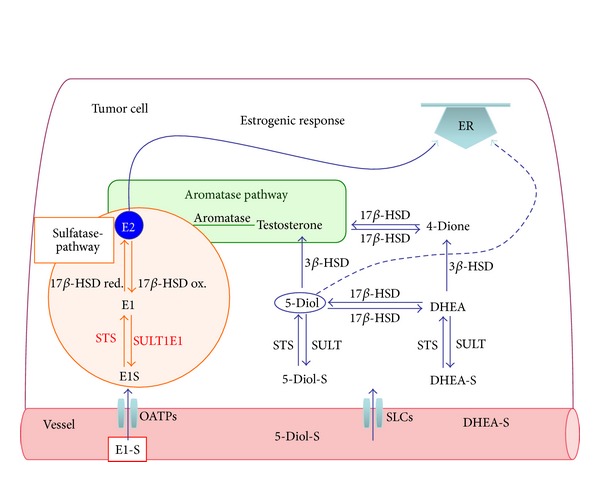
Estrone sulfate (E1S), androstenediol-sulfate (Adione-S), and dehydroepiandrosterone-sulfate (DHEA-S) are taken up into cells by organic anion transporting polypeptides (OATPs) and other transporters from the SLC-family. The “sulfatase pathway,” estrone-3-sulfate (E1S), is taken up by the cells and is activated by the removal of sulfate by the steroid sulfatase (STS). E1 is converted to the biological most active estrogen, 17beta-estradiol (E2), by reductive 17beta-hydroxysteroid dehydrogenases (17beta-HSDs). E2 binds and activates estrogen receptors. Vice versa, the conversion of E2 to less active E1 is catalysed by oxidative 17beta-HSDs. For inactivation, E1 is sulfonated by estrogen sulfotransferase SULT1E1 to E1S. The “aromatase pathway,” 5alpha-androstenediol-sulfat (Diol-S) and dehydroepiandrosterone (DHEA), are mainly derived from the circulation. Diol-S is converted 5alpha-androstenediol (5-Diol) by STS. It is converted into testosterone by 3beta-HSD. DHEA-S is hydrolyzed to form DHEA, which is further converted by 3beta-hydroxysteroid dehydrogenase to form androstenedione (4-Dione). Testosterone is formed by 17beta-HSD from 4-Dione. Testosterone is converted to E2 by the aromatase (CYP19). 5-Diol binds and activates estrogen receptors, but with lower affinity than E2 (see [20, 22]).

To create E2, E1S is taken up into the cells. There, after the removal of sulfate, E1 is reduced by reductive members of the superfamily of 17beta-hydroxysteroid dehydrogenases (17beta-HSDs) to form E2. Oxidative 17**β**-HSDs catalyze the conversion of E2 to E1. Reductive 17beta-HSDs also inactivate androgens and catalyze also the formation of other estrogens, for example, 5alpha-androstenediol. Since 17beta-HSDs modulate the concentration of active estrogens and androgens, inhibitors of these enzymes may be applied in cancer therapy [9, 10] (Figure 1).

**Figure 2:**
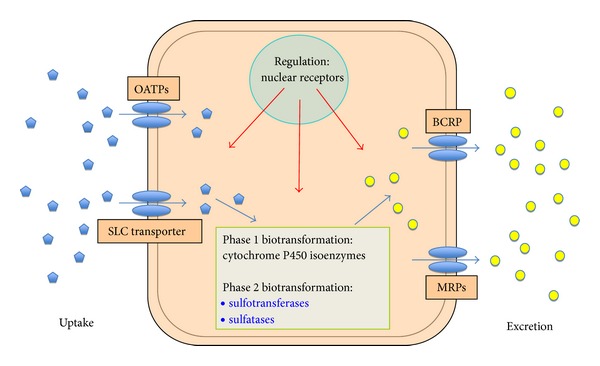
Uptake of E1S from the blood by organic anion transporting polypeptides (OATPs) and other SLC-Transporters. Biotransformation is mediated by phase 1 biotransformation (cytochrome P450 and isoenzymes) and phase 2 biotransformation (sulfotransferase and sulfatase). The excretion of sulfated estrogens is achieved by the breast cancer resistant protein (BCRP, ABCG2) and multidrug resistance related proteins (MRPs). Uptake, biotransformation and excretion is transcriptionally regulated by nuclear receptors, for example, the pregnane X receptor (PXR), which acts as a xenobiotic-activated transcription factor.

Polar estrogen sulfates, particularly, E1S, are taken up into cells by specific transport proteins from different families of SLC transporters including the family of organic anion transporters SLC21 or organic anion transporting polypeptides (OATPs). Within this concept, transporters from the OATP (SLC21) family such as OATP1A2, OATP1B3, OATP2B1, and OATP3A1 contribute to the cellular accumulation of E1S [11, 12], while ABC-efflux pumps from the MRP-family (ABCC1 and ABCC2), and the breast-cancer resistance protein (BCRP, ABCG2) mediates the efflux of E1S from the cells [13] (Figure 2). Uptake, biotransformation and excretion are transcriptionally regulated by nuclear receptors, for example, the pregnane X receptor. Furthermore, the variability in the expression levels and gene variants of transporters and enzymes can affect expression and function. These mechanisms may therefore influence the susceptibility of individuals to certain malignancies [14, 15].

As sulfated estrogens are unable to bind to the estrogen receptors, sulfonation of estrogens results in their inactivation. Therefore, conjugation with sulfate protects cells and tissues from an excess of active estrogens, and this may contribute to the prevention of hormone-dependent cancer cells. It further indicates that the balance between sulfate conjugation by the Phase 2 metabolizing enzyme estrogen sulfotransferases (SULT1E1) and the removal of the sulfate by the steroid sulfotransferase (STS) is important to store the hormone in an inactive form in the cells [16, 17].

Conjugation of lipophilic estrogens with sulfate is a main pathway for estrogen inactivation in estrogen target tissues. Sulfate conjugation of E2 is catalyzed by the Phase 2 drug metabolizing enzymes of the family of cytosolic sulfotransferases (SULTs) [18]. The isoform SULT1E1 is known as estrogen sulfotransferase, as it catalyzes the sulfonation of E1 and E2 with high efficiency at physiological concentrations. The sulfate conjugation of androgenic precursors, for example, dehydroepiandrosterone (DHEA), is mainly achieved by another SULT isoenzyme, namely, the SULT2A1 enzyme [18]. Both, 5alpha-androstenediol-sulfat (Diol-S) and dehydroepiandrosterone (DHEA) are mainly derived from the circulation. Diol-S is converted to 5alpha-androstenediol (5-Diol) by STS. It is converted into testosterone by 3beta-HSD. Dehydroepiandrosterone-sulfate (DHEA-S) is desulfonated to dehydroepiandrosterone (DHEA) and converted by 3beta-HSD to 4alpha-androstenedione (4-Dione), a precursor for testosterone formed by 17beta-HSD. Testosterone is converted to E2 by the aromatase (CYP19). 5-Diol binds and activates estrogen receptors, but with lower affinity than E2 [19]. 

**Figure 3:**
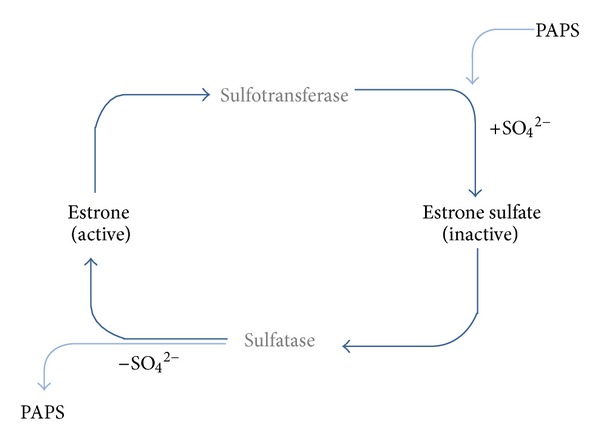
Conjugation of estrone (E1) with sulfate by the estrogen sulfotransferases (SULT) results in the formation of inactive estrone sulfate (E1S). Sulfated estrone is reactivated by the steroid sulfatase (STS) which catalyzes the removal of sulphate, forming estrone (E1). The sulfuryl group donor for the sulfotransferase-catalyzed sulfation is 3′-phosphoadenosine 5′-phosphosulfate (PAPS). The enzymatic reaction requires the acceptor (R-OH) and the donor PAPS to bind to a sulfotransferase. PAPS is synthesized by PAPS-synthesizing enzymes (PAPSS1 and PAPSS2). 3′-phosphoadenosine 5′-phosphosulfate synthase (PAPSS) catalyzes the biosynthesis of PAPS, which serves as the sulfate donor (see [22]).

As depicted in Figures 1 and 3, sulfonation of E2 forms inactive estradiol sulfate (E2S), which can be reactivated following removal of the sulfate by the cytosolic estrogen sulfatase STS. Sulfate (SO_4_^2−^) is obtained from the diet and the intracellular metabolism of sulfur-containing amino acids, including methionine and cysteine, and is an important nutrient for human growth and development. 

The sulfuryl group donor (cosubstrate) for the SULT-catalyzed reaction to add the sulfate moiety to hydroxyl groups is 3′-phosphoadenosine 5′-phosphosulfate (PAPS). The reaction products are sulfated estrogens and adenosine 3′, 5′-diphosphate (PAP). PAPS is generated by PAPS-synthesizing enzymes (PAPSS). Two isoforms, namely, PAPSS1 and PAPSS2, are known to be expressed in various tissues [20]. PAPSS1 might be important for growth of estrogen-sensitive breast cancer cells as a recent study revealed that overexpression of SULT1E1 and PAPSS1 resulted in growth inhibition [21]. 

## 2. Steroid Sulfatase (STS)

The steroid sulfatase (STS) belongs to the family of arylsulfatases in the sulfatase superfamily, whose members catalyze the hydrolysis of sulfate ester bonds in various endogenous and exogenous substrates.

STS is also known as arylsulfatase C, and in contrast to the cytosolic expression of arylsulfatases A and B, this enzyme is located in the endoplasmic reticulum of various tissues [23]. STS has a central role in the formation of active sex steroid hormones, as it hydrolyzes several steroid sulfates, including E1S and DHEA-S to E1 and DHEA, respectively [17]. 

The human STS gene is localized on the X-chromosome and consists of 10 exons. Inactivating mutations in STS gene have been associated with X-linked ichthyosis. Six different promoters were detected to drive STS expression giving rise to transcripts with unique first exons, and exon 1 alpha was associated with the promoter that drives expression in the placenta [24]. Induction of STS transcription by estradiol through binding to ER and via activation of estrogen-response elements in the promoter region results in driving the 1a and 1b transcripts in breast carcinoma [25]. Furthermore, regulation of STS activity by tumor necrosis factor alpha and interleukin 6 was found in breast cancer, most likely through a posttranslational modification [26]. 

## 3. Estrogen Sulfotransferase (SULT1E1)

Cytosolic sulfotransferases transfer sulfate from active sulfate (5′phosphadenosine-3′-phosphosulfate) to nucleophilic groups of their substrates. Belonging to the group of Phase 2 detoxification enzymes, they catalyze the biotransformation of hydroxysteroid and thyroid hormones, phenols, arylamines, and primary alcohols.

Four SULT families have been identified, namely, the phenol-metabolizing SULT1, the hydroxysteroid sulfating SULT2, and the SULT family 4 and 6 [18]. The two latter families are poorly characterized for their substrate specificity and tissue distribution.

At least six SULT isoforms catalyze the sulfate conjugation of E2, but only two, namely, SULT1E1 and SULT2A1 mediate the sulfonation of estrone (E1). 

SULT1E1 is considered as the “estrogen sulfotransferase,” as it has the highest affinity for E2 and E1 from all SULTs. It is the only SULT that displays an affinity for E1, E2, and various synthetic estrogens in a physiological concentration range (in the nanomolar range) [26]. Deletion of SULT1E1 genes results in reproductive abnormalities involving both male and female animals [27]. In the liver, the pregnane X receptor was found to represses the SULT1E1 gene, which may block inactivation of estrogens [28]. The SULT1E1 gene is located on chromosome 4q3.12, and its mRNA is detectable in a great variety of tissues. This would suggest that SULT1E1 may protect peripheral tissues from an excess of estrogens. Various SNPs has been detected in the human SULT1E1 gene, and some are linked to the recurrence of hormone-dependent cancer [29].

## 4. Enzymes in the Sulfatase Pathway in Estrogen-Associated Cancer

Data on the expression of enzymes in the sulfatase pathway in some estrogen-associated cancers are given in the following sections. Generally, the data on the expression of enzymes for the formation of E2 are rather inconsistent. This might be due to the fact that expression of enzymes in the estrogen metabolism and the concentration of circulating steroids are highly variable even in healthy persons, and they are even more varying in patients with cancer. Therefore, selection of patients with defined clinical parameters is important for studying these pathways. 

Cancer in a certain organ is not a uniform disease. A specific histological pattern and the molecular signature allow division of most hormone-dependent cancers into various subgroups. These are subgroups of cancer in a certain organ which have a different etiology and will produce a different response to a certain therapeutic regimen. However, even in a defined tumor type, there are great variations in the expression levels of different proteins in different tumor regions. This means that the expression in the tumor center can be completely different from that in one tumor front adjacent to the tumor center or in the front adjacent to the noncancerous tissue. 

So far, most studies were done in rather heterogeneous collectives of patients with a certain tumor in an organ. Also, assessment of target proteins by immunohistochemistry was mostly done on undefined tumor regions. This may explain the often conflicting data on the expression of enzymes and targets in molecular pathways [30].

### 4.1. Breast Cancer

Breast cancer remains the leading cause of cancer in woman worldwide. It occurs in both men and women, although male breast cancer is rare (approx. 1% of the rate in women) [30].In 2008, the estimated incidence of breast cancer in woman was 1,384.155 cases, and the mortality was 458.503 cases [31]. Estimated new cases and deaths from breast cancer in women are 226.800 and 39.510 women in the United States in 2012 [30].

More than 70% of breast cancers express ERs and progesterone receptors, PG-A and PG-B. Therefore, a major concern is whether or not the application of hormone replacement therapy (HRT) would increase the risk of breast cancer in postmenopausal women. According to the 2012 analysis published in the Cochrane Database Syst. Rev., hormone-replacement therapy with estrogens only did not increase the risk of breast cancer in postmenopausal women at a mean age of 60 years, but the combined continuous therapy with estrogens and progesterone-derivates significantly increased the risk for this cancer [32]. The breast cancer risk associated with HRT is higher for estrogen receptor-positive cancers than for estrogen receptor-negative cancers and for low-grade cancers compared with high-grade cancers. The increased risk of breast cancer dissipates within 2 years after finishing HRT [33].

50–70% of all invasive breast cancers are invasive ductal tumors, which arise in the milk ducts of the breast. According to the expression pattern of specific genes, cancers are further subdivided into four major molecular subtypes: luminal A, luminal B, triple negative/basal-like, and HER2 type tumors. Both luminal A and luminal B tumors express ERs, while the triple negative/basal-like tumors and HER2-type tumors are negative for ERs and PGs. Lobular carcinomas (10–20%) start from cells in the lobuli and can also be divided in these subtypes [34].

The luminal A breast cancer is the most common subtype, representing 50–60% of the total. It is characterized by the expression of ER targeted genes that are typically present in the luminal epithelium lining the mammary ducts, absence of HER2, a low proliferation rate, and a low histological grade. Based on their molecular profile, all cases of lobular carcinoma *in situ* and most of the infiltrating lobular carcinomas belong to this subtype. Luminal B molecular profile tumors (10%–20% of all breast cancers) are more aggressive, have a higher histological grade, and a worse prognosis [35].

Several data show that estrogens are enriched in breast cancer tissue as compared to normal tissue. They surplus the plasma levels by 23-fold in women at reproductive age and 23-fold in postmenopausal patients. In older women, nearly all E2 is locally produced, but also in younger women up to 75% originate from the local production [35]. In breast cancer, the STS pathway with the reduction of E1 to E2 is catalyzed by reductive 17beta-HSDs. This is the most prominent pathway and prevail the aromatase pathway with estrogen production from testosterone and its precursors by 50–200-fold [6]. Indeed, many studies showed that STS activity is much higher than aromatase activity in breast tumors, the activity of the enzyme is also higher in the carcinoma than in the nonmalignant tissue, and expression of tissue-specific transcripts of STS is controlled by ERalpha signaling in normal and cancerous breast tissue [36]. Studies in patients with ERalpha-positive breast cancer showed that expression of more active STS isoforms under estrogen therapy may cause upregulation of E2, which would further promote cancer progression [36]. Moreover, high levels of STS mRNA expression in tumors are associated with a poor prognosis [37]. 

Breast tumors expressing ERs may benefit from adjuvant endocrine therapy with antiestrogens such as tamoxifen, which is applied in pre- and postmenopausal women. In postmenopausal women blocking the estrogen production by inhibitors of estrogen formation, for example, aromatase inhibitors is an effective therapy for cancer prevention [38, 39]. But some tumors are intrinsically resistant against endocrine therapy, or others acquire resistance against hormonal treatment later. STS and 17beta-HSDs in local estrogen production provide novel potential targets for endocrine therapy [10, 40]. Therefore, the development of combined of STS/aromatase inhibitors and STS/17 beta-HSD type 1 inhibitors will be required in the future. 

### 4.2. Endometrial Carcinoma

Endometrial carcinoma is the most frequent gynecological malignancy in other in industrialized nation including the USA. 47.130 new cases and 8.010 deaths from endometrial cancer in the United States are estimated for 2012. In 90% of all cases, endometrial carcinomas occur sporadic. Most endometrial cancers are adenocarcinomas. They are subclassified into type 1 or type 2 tumors. Type 1 tumors (80% of all sporadic cases) are found in pre- and postmenopausal women and develop from precursor lesions (hyperplasia, intraepithelial neoplasia) through excessive stimulation by estrogens, if it is either not counteracted by progesterons or lasts over a prolonged time. Data from the 100 Million women study showed that estrogens increase the risk of endometrial cancer, while progestagens counteract the adverse effect of estrogens on the endometrium in women with a mean age of sixty. Because estrogens stimulate the proliferation and progesterons the differentiation of endometrial cells, continuous HRT with the estrogen-progestagen combination will reduce the risk of these carcinomas, which are sensitive to these hormones [41, 42].

Two major subtypes of endometrial carcinomas can be discriminated. In type 1 tumors, PTEN gene silencing together with defects in DNA mismatch repair genes and/or mutations in the K-ras and/or beta-catenin genes are frequently present and contribute to the malignant transformation via hyperplasia, intraepithelial neoplasia, and to the carcinoma. These type 1 endometrioid endometrial cancers are well differentiated and estrogen sensitive. Type 2 tumors develop either *de novo* or from metaplasia to serous-papillary or clear-cell carcinomas. They carry mutations in TP53 and Her-2/neu and seem to arise from a background of atrophic endometrium [43]. Overall, type 1 tumors have usually a better prognosis than high grade, estrogen-independent type 2 tumors [44]. 

In the endometrium, ERalpha and ERbeta are expressed, and as shown for other hormone-dependent tumors, ERalpha levels are higher than that of ERbeta. Since ERbeta is considered to have antiproliferative and proapoptotic effects, it may act as repressor for ERalpha. If ERbeta is reduced, E2 would rather act through ERalpha signaling.

Indeed, many studies showed that the receptors are differently expressed in normal and cancerous endometrium, but results are controversial. Higher, lower, and no changes in ratio between ERalpha and ERbeta were reported [45, 46]. Similar to the data from breast cancer, the levels of E2, E1, and E1S were found to be higher in cancer patients than in healthy postmenopausal women. Highest levels are seen for E1 [47]. Furthermore, the concentrations of estrogens are several times higher in the cancerous endometrium than in the surrounding normal tissue [48].

Since the majority of the endometrial cancer patients are postmenopausal women, local formation of E2 from circulating precursors either from circulating androgens via the aromatase pathway or from E1S via the sulfatase pathway becomes important. Data on the expression of aromatase in endometrial cancer are rather inconsistent. Although aromatase inhibitors have become the gold standard for endocrine treatments in the postmenopausal patients with estrogen-dependent breast carcinoma, the therapeutic value of aromatase-inhibitors in estrogen-sensitive endometrioid carcinoma is also not clear [49].

Regarding aromatase expression in endometrial cancer, early studies [50, 51] showed that mRNA levels and the activity of the enzyme are higher in endometrial carcinomas than in the normal endometrium. It was demonstrated that aromatase is mainly located in stromal cells rather than in cancer cells. Interactions between stroma and tumor cells will provide E2 for the proliferation of cancer cells. This was shown in a coculture of Ishikawa cells (an endometrial carcinoma cell line) with stromal cells [52]. In a more recent study, aromatase mRNA expression was shown to be present in peritumoral tissue but not in the endometrial cancer [47]. In another study, aromatase was higher expressed in well-differentiated tumors than in normal tissue and in high grade tumors. However, overall aromatase mRNA levels in the endometrial carcinomas were shown to be low [53]. In line with these findings, only weak staining for aromatase was seen in cancerous endometrium [54]. In the latter study, no significant differences in aromatase mRNA expression levels between cancerous and adjacent normal tissues were seen. However, in some specimens from endometrial cancer, 17beta-HSD (AKR1C3) active to form testosterone from androstenedione was upregulated. This may increase testosterone for conversion to E2 by aromatase, and its may act as an estrogenic 17beta-HSD to produce E2 from E1. All enzymes necessary for intracrine production of E2 via the sulfatase pathway, namely, STS, reductive 17beta-HSD type 1,5,7,12, and oxidative 17beta-HSD type 2,4,8 are expressed in these tumors. These reductive 17beta-HSDs are thought to convert E1 to E2, and vice versa, oxidative 17beta-HSD isoenzymes to form E1 from E2 [54, 55]. The study of Lépine et al. [47] showed that 17beta-HSD enzymes, which convert E1 to E2, are highly expressed in normal tissue and are even higher in tumors. Additionally to the levels of 17beta-HSD isoenzymes, also levels of the sulfatase STS are increased. STS actives E1S, as it removes the sulfate group. In summary, this leads to an increase of levels of active estrogens in endometrial tumors [56]. 

Also SULT1E1, which inactivates E2 by producing E2S, is weakly expressed in these tumors. Utsunomiya et al. [57] demonstrated by immunohistochemistry that SULT1E1 is expressed in normal endometrium during the secretory phase in the menstruation cycle. In the majority of tumors, SULT1E1 levels were reduced, while STS levels were high. 

### 4.3. Ovarian Carcinoma

Ovarian carcinoma that is the fifth most common cancer among women in Western countries is the most deadly gynecological malignancy. In 2012 in the USA, there are 22.380 estimated new cases and 15.500 deaths [30]. The estimate incidence of ovarian cancer worldwide was 224.747 cases in 2008 [31].

Ovarian carcinomas are now known as heterogeneous tumors. It is currently accepted that only gonadal, stromal tumors, and germ cell tumors (5% of all ovarian carcinomas) are tumors of cells present in the normal ovary. The great majority of the ovarian carcinomas develop in cells from outside the ovary, and involvement of the ovary is secondary [58–60]. Based on histopathological characteristics and the distinct molecular signature, five types of ovarian carcinomas that account for over 95% of all cases can be discriminated: high-grade serous carcinomas (HGSC), low-grade serous carcinomas (LGSC), endometrioid, clear-cell, and mucinous ovarian carcinomas [60]. Endometrioid (10%) and clear-cell (10%) carcinomas originate from endometriosis in the ovary, and HGSC and LGSC were previously thought to develop from the ovarian surface epithelium [61], but it is now agreed that they develop from the tubal epithelium in an independent way using different molecular pathways [60]. The most frequent HGSC (70–80% of all ovarian carcinomas) may arise from precursor lesions in the epithelial cells in the distal fimbriated end of the fallopian tube or the implantation of tubal-type epithelium into the ovary. SLGCs (5%) are associated with a serous borderline component. While HGSCs have a bad prognosis, LGSCs have a better outcome [62]. One reason is that because of absence of specific symptoms, HGSCS is usually detected at an advanced stage, in which the cancer has spread within the pelvis. In these cases, the five-year survival rate is less than 40%. Although HGSCs are initially sensitive to chemotherapy, they become resistant within a short period. TP 53 mutations are typically present in HGSCs, and mutations in BRAF, KRAS are characteristically found in LGSCs. Women with BRCA1/2 germline mutations are at high-risk factors for HGSCs (10% of all cases) [63]. Data on the expression of ERs and (PGs), whether they may serve as predictive biomarker for these tumors, are rather controversial, and only few studies discriminate between different tumor types. There is increasing evidence that ERalpha induces proliferation of ovarian cancer cell growth, whereas ERbeta has been described to mediate proapoptotic and antiproliferative effects. PR-A is a transcriptional inhibitor of ERalpha, and PR-B induces of cell differentiation. These four steroid hormone receptors were found to be commonly expressed in LGSCs, but their expression rate was significant reduced in HGSCs [64]. Recent epidemiological data showed that in patients with HGSCs, expression of ERs and PG-B receptor was associated with a favourable outcome as analysed by univariate analysis. In the multivariate analysis, only PR-B was an independent prognostic marker for the patient survival [65].

Steroid hormones may play a role in the development of sporadic ovarian cancer. While oral contraceptive have a protective effect, hormone replacement therapy with estrogen only or in combination with progesterones may increase the risk of ovarian cancer. In the 100 million women study, the risks associated with HRT varied significantly according to the tumor histological type. In women with epithelial tumors, the relative risk for current versus never use of HRT was greater for serous than for mucinous, endometrioid, or clear-cell tumors [66]. Data from a recent study in a large cohort of women (909.946 cases) in Denmark revealed that hormone users had higher risk of serous and endometrioid type cancers, but not of ovarian cancer of the mucinous and clear-cell type [67]. Compared with never users, women taking unopposed estrogen therapy had increased risks of both serous tumors and endometrioid tumors but decreased risk of mucinous tumors. Similar increased risks of serous and endometrioid tumors were found with estrogen/progestin therapy. Consistent with results from other studies [66], the authors found that ovarian cancer risk varied according to tumor histology [67].

In most studies on the expression of steroid hormone receptors and on the expression of enzymes involved in the local estrogen synthesis in ovarian cancers cells, there is no discrimination between different types of ovarian cancer. 

The aromatase pathway is active in ovarian cancer, but so far clinical studies using antiestrogens or aromatase inhibitors were rather disappointing [68]. However, recent data suggest that endocrine therapy might benefit women with certain cancer subtypes. For example, women with recurrent LGSC and expression of ER, application of hormonal therapy might be of benefit [69]. Furthermore, aromatase inhibitors were found to be promising in the treatment of rare granulose tumors in the ovary [70]. 

**Figure 4:**
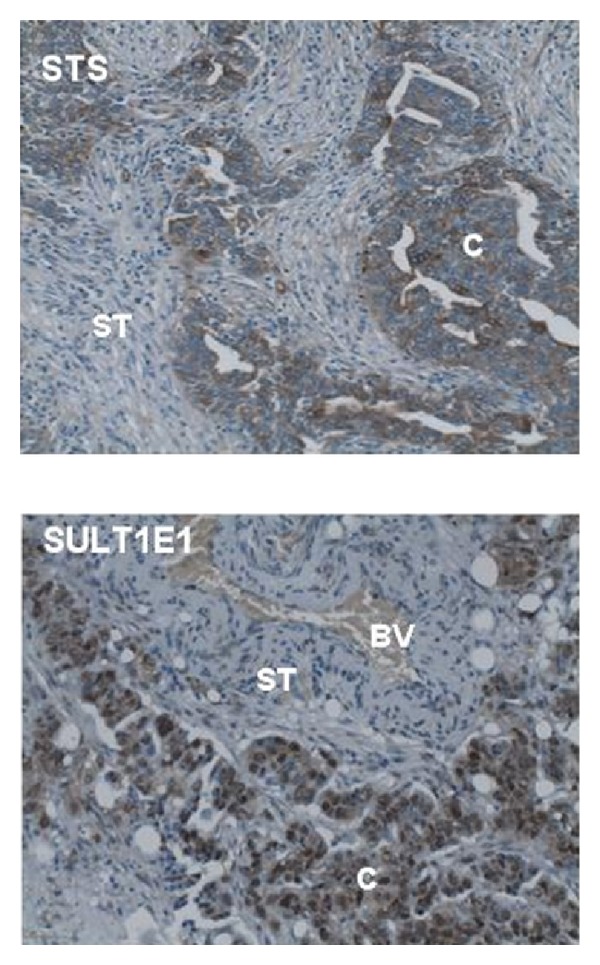
STS (steroid sulfatase) and SULT1E1 (estrogen sulfotransferase 1E1) in high-grade serous ovarian carcinoma. Immunoreactivity of STS and SULT1E1 is demonstrated in paraffin-embedded tissue sections from ovarian carcinoma. Sections were probed with an antibody against STS (GTX 105498, GeneTex, Irvine, CA) and SULT1E1 (NBP1-56977, Novus Biol., Littleton, CO), respectively. STS and SULT1E immunoreactivity is visible in the cancer cells (C). ST = tumor stroma, BV = blood vessel.

Intracrine production of E2 through the sulfatase pathway from E1S may be of particular interest for the diagnosis and treatment of ovarian cancer in postmenopausal women, although formation of E2 from circulating estrogen sulfates occurs in younger women as well. 17beta-DSH type 1 and 5 and STS were previously detected in samples from ovarian cancer patients at the mRNA and protein levels [71–73]. Steroid sulfatase enzymatic activity was determined [74]. STS was detected in ovarian surface epithelium and granulosa cells. In an immunohistochemical study, STS was detected in 30% of serous and 50% of mucinous adenocarcinoma specimens [75]. Also studies in our lab show high levels of STS and moderate to low expression of SULT1E1 in a collective of patients with advanced ovarian cancer (Figure 4).

Further studies in estrogen receptor alpha-expressing OVCAR-3 cells showed that STS is inhibited by the STS inhibitor STX64. As STS expression is highly variable and found at high levels in tumors of nearly all patients, blocking the sulfatase pathway may be of values for these patients [75]. Also the aromatase pathway to convert the androgens to estrogen is active in ovarian cancer cells and will lead via the conversion of dehydroepiandrostenedione to androstenedione to the production of E2. Therefore, a combined inhibitor for both, STS and aromatase, might be suitable for these patients [76].

### 4.4. Colorectal Cancer

Estimated new cases and deaths from colon and rectal cancer in the USA, in 2012, were 103.170 new cases of colon cancer and 40.290 cases of rectal cancer. 51.690 deaths were from colorectal cancer [30]. These cancers accounts for approx. 10% of new cancer diagnoses among women worldwide with an incidence of 571.204 cases and a mortality of 288.654 worldwide [31]. Colorectal cancer is the third leading cause of cancer for women after lung and breast cancer. Screening programmes for colorectal cancer in man and woman over the age of 50, now widely applied in many industrialized countries, are leading to a reduction in the incidence and mortality of colorectal cancer (similar to data shown for the USA) [77].

Estrogens were found to play a role in the pathogenesis of colorectal carcinomas and may have a protective role [78]. Many epidemiological studies have found a reduction in the risk of colon cancer associated with use of estrogen/progesterone-based regimens of HRT. Although overall diagnoses were decreased, a larger proportion of poor prognosis tumors was detected among these patients [79]. In the estrogen-alone group, there was no reduction in the risk of colorectal cancer. Therefore, a recent evaluation of the outcome of various studies on HRT led to the conclusion that application of any HRT regimen to prevent colorectal cancer is not recommended [80].

In many colon carcinoma specimens and colon cancer cell lines, ERbeta [81], aromatase, STS, SULT1E1 [82], and 17**β**HSDs [83] are expressed. It was also demonstrated that concentrations of estrogens in the cancer tissue were twice of those in normal colonic mucosa [82]. Moreover, higher intratumoral concentrations of total estrogens were significantly associated with poorer survival. Thereby, the ratio between STS and SULT1E1 will determine the intratumoral concentration of total estrogens and determine the clinical outcome of the patients. However, these findings are not fully supported by epidemiological data on the application of estrogens to prevent colon cancer (see above). 

Other findings would support the beneficial effects of estrogens. The gene coding for 17beta-HSD1 was found to be reduced by promoter methylation in colon cancer. This will reduce the formation of E2 from E1 via this 17beta-HSD [84]. Expression of type 2 and 4 isoenzymes of the 17beta-HSD family was also shown to be significantly decreased in tumors compared to normal mucosa [85]. Importantly, downregulation of ERbeta was found to be associated with a poor prognosis in the patients [86, 87].

### 4.5. Estrogen Sulfates in Metabolic Disease Related to Cancer

The incidences of breast cancer as well as of the metabolic syndrome with obesity, insulin resistance, hyper-insulinemia, high blood pressure, and type 2 diabetes have increased over the past decades in industrialized countries. The loss of the sensitivity of cells to insulin is associated with changes in the signaling of chemokines, cytokines, growth hormones, and steroid hormones [88–90]. This may explain why metabolic disease goes along with an increased risk of certain cancers, for example, breast and colon cancer. Studies in patients with the metabolic syndrome showed that levels of SULT1E1 for the inactivation of estrogens correlate with the expression of proinflammatory factors. The risk appears to be higher in postmenopausal than in premenopausal women, suggesting the importance of intracrine estrogen formation [89, 90]. Although there is sufficient evidence for a relation between metabolic syndrome and certain cancers, the exact molecular mechanism for the metabolic syndrome in the carcinogenesis is not thoroughly understood yet. Nevertheless, various potential direct and indirect links exist between obesity, metabolic syndrome, type 2 diabetes, and an increased risk of colon cancer. Modification of insulin and insulin-like growth factors pathway, leptin signaling, adipose-tissue induced changes in estrogens and androgens, and inflammatory molecules may contribute [90].

It is well known that E2 is an important regulator of the energy balance and metabolic homeostasis not only in women but also in men [91]. In postmenopausal women, low circulating estrogen levels lead to accumulation of visceral fat, insulin resistance/glucose intolerance, and osteoporosis [92]. As estrogen promotes the differentiation of bone marrow-derived mesenchymal stem cells to bone-building osteoblasts, low estrogen levels will favor adipocyte formation. Differentiation of adipocytes is reduced by SULT1E1 [93]. As a consequence, decreasing estrogen levels is associated with a decreased bone mass and accumulation of fat [94]. Similar changes are observed in men with estrogen deficiency or during ageing with declining levels of steroid hormones. 

Local estrogen synthesis is also effectively carried out in adipocytes and human bone cells. E1S is a major source of local bioactive estrogen formation [95]. Also, SULT1E1 is also expressed at higher rate in malignant bone tumors than in benign ones [96]. In adipocytes, all enzymes important for the local formation of estrogen are expressed, and their levels increase after adipocyte differentiation [97]. In SULT1E1 knock-out mice, loss of SULT1E1 causing an excess of estrogens leads to the formation of smaller patches from white fat and insulin resistance [97].

In type 2 diabetes, induction of hepatic SULT1E1 is most frequently observed. Loss of SULT1E1 improves the metabolic function in a female mouse model of type 2 diabetes, restores insulin sensitivity, and blocks hepatic gluconeogenesis and lipogenesis [98]. Since in diabetes, upregulation of SULT1E1 decreases E2 levels, inactivation of the enzyme will prevent loss of estrogens and normalize estrogenic activity in the liver. This beneficial effects of SULT1E1 inactivation were absent in ovariectomized mice. These effects were also sex specific, as SULT1E1 loss in males worsened the diabetic phenotype and led to a decreased islet beta-cell mass, failure of glucose-stimulated insulin secretion, increased macrophage infiltration, and inflammation in white adipose tissue. The authors suggest that inhibition of SULT1E1 at least in females may represent a novel approach in the therapy of type 2 diabetes [98, 99]. However, it has to be considered that type 2 diabetes mostly occurs in women after the menopause when local formation of steroid hormones from adrenal precursors becomes important. Since extragonadal estrogen production is typical for primates [2], the benefit of increasing levels of active estrogens by reducing SULT1E1 may have to be studied in a proper model for type 2 diabetes in this group. In any case, higher estrogen levels are thought to have beneficial effects on type 2 diabetes, but the risk of the induction of hormone-sensitive cancers may be considered as well.

## 5. Steroid Sulfatase Inhibitors as Agents for a Therapy of Hormone-Sensitive Tumors

Hormone therapy is used to treat both early and advanced breast cancer and to prevent breast cancer in women who are at high-risk of developing the disease. Currently, the most widely used therapies for the treatment of hormone-dependent cancer is to block the action of steroid hormones. Adjuvant endocrine therapy with the selective estrogen receptor modulator (SERM) tamoxifen is recommended for premenopausal women with a history of atypical hyperplasia to reduce breast cancer risk. Raloxifene, another selective estrogen receptor modulator, was found to be equivalent to tamoxifen in reducing the risk of developing invasive breast cancer in postmenopausal women. However, it did not provide the same level of risk reduction for developing noninvasive breast cancer. Aromatase inhibitors, which block the conversion of androstenedione to estrone, are applied in postmenopausal women. Currently, third-generation aromatase inhibitors, which comprise the nonsteroidal compounds anastrozole and letrozole, and the steroidal exemestane are finding widespread application in the clinic (for reviews see [100, 101]). However, the development of resistance to the endocrine therapy is still a major therapeutic problem and limits the clinical benefit of their application. 

Regarding the fact that local formation of E2 from E1S via the sulfatase pathway is more effective in some hormone-dependent tumors than formation of E2 via the aromatase pathway [102], STS inhibitors offer an attractive strategy to reduce estrogenic stimulation of hormone-sensitive tumors [103]. Furthermore, high levels of STS and low SULT1E1 expression are regarded as prognostic factors in hormone-sensitive cancer, for example, of the breast. Blocking STS may therefore offer an additional benefit in the therapy, and STS inhibitors are under development [104, 105].

The first approach was to block the desulfonation of E1S by offering nonhydrolysable E1S analogues, for example, sulfates of the flavonoid daidzein. However, these compounds possess high intrinsic estrogenic activity. Therefore, different STS inhibitors have been developed, a number of successful products in which the sulfate moiety was replaced by a sulfamate, for example, estrone 3-o-sulfamate were introduced, and estradiol 3-sulfamate was introduced into clinical trials but failed because of the estrogenic effects of the products. To prevent the estrogenic effects, sulfamate-based nonsteroidal inhibitors were introduced, and the most successful derivate was the cyclopentane carboxylate derivative STX64 (irosustat), which is present in clinical development (Phase 2 clinical trials) for the treatment of patients with advanced breast cancer and other hormone-dependent cancer. The structure is a tricyclic coumarin-based sulfamate. It undergoes desulfonylation as a result of its mechanism of STS inhibition [104].

Regarding the benefit of the therapeutic application of aromatase inhibitors and present knowledge on the importance of the inhibition of STS, compounds to inhibit both pathways (so-called DASIs) are now under investigation. They may provide a new therapeutic concept. One approach to create such DASIs is the insertion of a pharmacophore for STS inhibition into an established aromatase inhibitor, for example, letrozole. For example, the pharmacophore for STS inhibition, a phenol sulfamate ester, and the pharmacophore for aromatase inhibition, an N-containing heterocyclic ring, are incorporated into a single molecule. Another group of DASIs comprises derivatives of a known STS inhibitor incorporating a heme-ligating heterocyclic ring [105]. Many of these novel inhibitors of both enzymes were found to be effective in preclinical studies. This approach offers the opportunity for further continuing preclinical development of such dual inhibitors. 

## 6. Steroid Sulfatase as a Target for Biomedical Positron Emission Tomography Imaging 

Positron emission tomography (PET) is a biomedical imaging technique in which compounds labelled with positron emitting radioisotopes, for example, ^1^1C, ^18^F, are applied to monitor processes in cells. For PET, trace amounts of positron-emitting radionuclide-labelled compounds are retained in cells in different tissues either because of their binding to specific receptors or by being taken up into cells by specific transmembrane transporters where they undergo an enzyme-catalyzed conversion. As PET provides tomographic images of the distribution of the radioactive traces in tissues, the technique is widely used to diagnose cancer and cancer metastasis [106], and multitargeted anticancer agents are now developed as enzyme-based cancer imaging agents. For breast cancer diagnosis, STS catalyzing the hydrolysis of steroid sulfates to estrogens is an attractive target, and this is also true for aromatase. To target both enzymes, ^11^C-labelled sulfamate derivatives were designed as potential PET dual aromatase-steroid sulfatase inhibitor (DASSI) radiotracers [107]. Another enzyme, which is highly expressed in a great variety of tumors, is carbonic anhydrase 2 (CA2), and recently a bis(sulfamoyl)estradiol derivative, which functions as a dual-function STS-CA2 inhibitor, was developed. This compound has a high antiproliferative potential in many tumor cells [108]. Additionally, antiangiogenic effects were shown *in vitro* and *in vivo,* and it may therefore be a good candidate for cancer treatment and molecular imaging of cancer.

## 7. Summary and Conclusion

Circulating inactive steroids in estrogen-dependent tumors are converted to the biological most active estrogen, 17beta-estradiol in the sulfatase, and aromatase pathway. In the sulfate pathway, estrone-3-sulfate (E1S) is desulfonated by steroid sulfatase (STS) to estrone (E1). Estrogens are inversely inactivated by sulfonation via the estrogene sulfotransferase (SULT)1E1 to the sulfated estrogens. E1 is converted to E2 by 17beta-hydroxsteroid dehydrogenases (17beta-HSDs) and vice versa. In the aromatase pathway, E1 and E2 are synthesized from the circulating precursors androstenedione and testosterone, respectively. The mechanism for the uptake and production of biological active steroids at extragonadal sites is described with the term “intracrinology.” Importantly, the *in situ* formation of E2 at the sites of their actions will influence the growth and progression of hormone-dependent tumors. This paper gives an overview about expression and function of enzymes of the sulfatase pathway, particularly of STS, in breast, endometrial, ovarian, and colorectal cancer. High expression of STS together with the overexpression of 17beta-HSDs may lead to an increased production of active E2. Higher levels of E2 and other active estrogens can result in the stimulation of tumor growth and progression of hormone-sensitive tumors of the breast, endometrium, and ovary. Altered sulfonation of estrogens is also implicated in the pathogenesis of the metabolic syndrome and type 2 diabetes. Here, the increased secretion of proinflammatory cytokines and chemokines by metabolic disturbed cells seems to contribute to carcinogenesis. Indeed, these diseases share common risk factors with cancers of the breast and ovary. Because in hormone-sensitive tumors, for example, breast cancer, estrogen formation by the sulfatase pathway exceeds that of the aromatase pathway by several folds (50–100-fold), blocking the sulfatase pathway should reduce the growth of estrogen-sensitive cancer. Various inhibitors of sulfate-removing STS were synthesized and offer a promising therapeutic approach to combat estrogen-sensitive tumors, particularly, if these compounds also inhibit enzymes of other cancer progression pathways (aromatase, carboanhydrase 2). One compound STX-64, lacking estrogenic effects, is currently undergoing clinical trials. Furthermore STS inhibitors might also be suitable as enzyme-based cancer imaging agents applied in the biomedical imaging technique positron emission tomography for the diagnosis and therapy of estrogen-sensitive cancer.
